# Power dynamics as a determinant of access and utilization of nutrition services by pregnant and lactating adolescent girls in Trans-Mara East Sub-County, Narok County, Kenya

**DOI:** 10.1186/s12889-020-08690-w

**Published:** 2020-04-19

**Authors:** Charles O. Opiyo, David Omondi Okeyo, Sussy Gumo, Elly O. Munde, Zablon O. Omungo, Maureen Olyaro, Rachel K. Ndirangu, Nanlop Ogbureke, Sophie Efange, Collins Ouma

**Affiliations:** 1 Christian Aid-UK, P. O. Box 13864-00800, Nairobi, Kenya; 2 Kenya Nutritionists and Dieticians Institute, P. O. Box 20436-00100, Nairobi, Kenya; 3 School of Arts and Social Science, Department of Religion, Theology and Philosophy, Maseno University, Maseno, Kenya; 4 School of Public Health and Community Development, Department of Biomedical Science and Technology, Maseno University, Maseno, Kenya; 5 Christian Aid-UK, 35 Lower Marsh, London, SE1 7RL UK

**Keywords:** Power dynamics, Nutrition, Lactating, Pregnant, Adolescent

## Abstract

**Background:**

During pregnancy or lactating, adequate nutrition for adolescents becomes critical to reduce risks for both child and maternal-related morbidity and mortality. Power dynamics play a massive role in health outcomes. The main objective of this study was to examine the power dynamics in the families and communities and their impact on the pregnant and lactating adolescent girls’ access and utilization of nutrition services in Trans-Mara East Sub-County, Narok County.

**Methods:**

A cross-sectional approach that employed mixed methods with both quantitative and qualitative research was adopted. Probability proportionate to size sampling techniques using cluster and simple random methods were used to practically access pregnant or lactating adolescents. Data was collected using questionnaires, in-depth interview and Focus Group Discussion. Quantitative data was analyzed descriptively using frequencies and inferentially using odds ratio and Z-test. Framework analysis was employed to analyze qualitative data. *P* ≤ 0.05 was considered statistically significant.

**Results:**

In the power dynamics analyses, the intrinsic capability (Intrinsic capabilities are those adolescent driven initiatives that facilitate their access to nutrition services) was more likely to decrease awareness by half (OR = 0.52, 95% CI = 0.4–0.7, *P* < 0.01) whereas extrinsic dependency was likely to increase utilization by 1.2 times (OR = 1.2, 95% CI = 1.0–1.5, *P* = 0.055). From the stakeholder power matrix, the health personnel had observable visible power to influence access and utilization of nutrition services. Additional results revealed that adolescents who draw their support from significant others were more likely to utilize nutrition services as compared to those who attempted to make their own efforts to seek these services. Furthermore, health personnel have the most influential powers in ensuring adolescents access services and thus the most important actors in the stakeholder matrix. Other actors requiring focus included parents, political figures and governments while stakeholder engagement have higher potential of increasing access and utilization of services through dialogue.

**Conclusions:**

Community access to nutritional services can be increased through use of multiple avenues to reach adolescents, including school-based, health system-based, community-based approaches and even marriage registries. A heightened engagement in the identified stakeholder network is necessary when planning community conversations, to ensure a multi-stakeholder approaches in meeting the nutrition needs of adolescents.

## Background

Worldwide, there is partial data explicitly on access and utilization of nutritional advice and services by adolescents. Use and utilization of nutrition services among adolescents are highly related to self-esteem, poverty, health beliefs, customs of the community in which they belong, the social structure and the level of education. It is well established that power dynamics, and specifically gender, play a massive role in health outcomes [1]. Disparities often occur on the basis of who has more power to either positively or negatively influence or prevent them to access and use the necessary services to enhance health [2]. For example, it has been shown that the power to augment or thwart an adolescent from accessing and utilizing nutrition services and pieces of advice are dictated by the community’s view on the different roles played by the different stakeholders [3]. Gender norms in any given society can lead to differences between males and females in regards to social position and power to access and utilize nutrition and other health-related services. Besides, one’s ability to make choices and act can materialize if an individual believes in self-worth and can conquer the fear of failure irrespective of external influences [4]. Several studies show that adolescent girls in low- and middle-income countries face multiple challenges including inadequate access to education, early marriage, low social position in the society and poor nutrition [5, 6].

In most communities in developing countries, it is often the role of a man at a household level to control decisions about the health of the family and even use of health services for the family [7]. A study carried out in Senegal have established that women’s ability to participate in decision-making transforms into more access and utilization of maternal and child health services [8]. Low social status in the society and restricted opportunities for livelihood, failure to train adolescents on matters pertaining to sexual health and decision-making undermines the ability of girls and women to take necessary action and engage in healthy behaviors or even seek healthy alternatives. Expansion of the ability of the adolescent to decide and act on the decision made affecting health-related aspects can be intensively addressed by reflecting on socio-economic and power inequalities in the context in which this capacity is involved. Boosting the financial status of girls and women through aspects such as micro-credit activities can empower them to improve their own, their children’s and at large, the household’s nutritional status [7].

Previous investigators compare distance and cost as factors that predetermine the decision of an adolescent to seek and utilize nutrition services and pieces of advice [9]. Their work reveals that the quality of care is so often prioritized when compared to cost. Nevertheless, they point out that the nature of service delivery to the adolescent is important, especially in regards to who and how they are served. Due to stigma and fear of being recognized as pregnant, most adolescents end up remaining silent and become more vulnerable to the prevailing conditions. These three components; cost, quality of care and distance to a health facility can give an indication on the possibility to decide and seek nutrition services [3, 10]. Pregnant or lactating adolescents who may make timely decision to seek nutrition care and support may face delays because the accessibility of nutrition and other health services is a major problem in many developing countries [11].

In Narok County, 40% of girls aged 15–19 years have begun child-bearing, almost two times higher than the Kenyan national level (18%), yet to date, no study has assessed the power dynamics and their impact on access and utilization of nutritional advice and services by adolescents in Trans-Mara East Sub-County, Narok County. It is against this background that the current study examined the power dynamics in the families and communities and their impact on access to nutrition services by the pregnant and lactating adolescent girls’ in Trans-Mara East Sub-County, Narok County, Kenya.

## Methods

### Study setting and research design

This study was conducted within Narok County where 40% of girls aged 15–19 years have begun child-bearing. In this region of study, it was established that 7.4% of adolescents are pregnant with their first child and 33% have ever given birth as compared to the national levels of 3.4 and 14.7%, respectively. These statistics are supported by the risks facing adolescents in Kenya include but are not limited to: high HIV infections, particularly among girls (16% of people living with HIV are aged 10–24 years); high rates of teenage pregnancies (18%); early marriages (11%) for older adolescents (15–19 years); persistent female genital mutilation (11%); high rates of anaemia (41%) among pregnant adolescents; high number of adolescents exposed to sexual violence (11%) and physical violence (50%) as well as low secondary school attendance with a net ratio of 47%. All these risks perpetuate further the vulnerability of this age group to a healthy life.

The study was carried out in Trans Mara East Sub-County within Narok County. Trans Mara East Sub-County was purposively selected since it is the smallest in size (275.4 km^2^), among the four sub-counties in Narok County and had the highest prevalence of teenage pregnancies based on previous survey (Christian Aid, 2018; *pers. comm.*). To achieve the objectives of the study, a cross-sectional approach employing concurrent mixed methods with both quantitative and qualitative research techniques was applied.

### Study population and sampling technique

#### Study population

The primary study population comprised of pregnant and lactating adolescent girls (aged 10–19 years old) resident in Trans Mara East Sub-County, assuming that the prevalence of pregnant and lactating mothers was 50% within the entire Trans Mara East Sub-County, from which a sample was drawn.

#### Sample size determination for quantitative approach

We initially determined sample size using the Cochran formula [12], which allowed for calculation of an ideal sample size given a desired level of precision, desired confidence level, and the estimated proportion of the attribute present in the population. A total sample size of 292 was applied as previously reported in our work [13]. Proportionate distribution was done across 25 clusters equivalent to villages and by adolescent status (i.e. pregnant or lactating). It is from these clusters that the adolescents for power analyses were drawn from.

#### Test for sample size adequacy

Based on the above formula, the minimum sample size at 90% confidence was 292 pregnant and lactating adolescents. However, given the nature of the questionnaire where 90% of key variable measures were based on 5 point-Likert scale, descriptive test for sample size adequacy using Kaiser-Mayor Olkin and Batt-test of sphericity was performed as previously described [13].

#### Sampling procedure

Cluster sampling was appropriate under the assumption given the existing ward and villages. Probability sampling techniques using cluster and simple random methods was used to practically access adolescents who were either pregnant or lactating as has been previously described [13].

### Methods of data collection

Quantitative data was collected using adolescent questionnaire targeting critical indicators of access, utilization and individual power dynamics. Focus Group Discussion (FGD) guide was administered to adolescents, their fathers and spouses, their mothers, Community Health Volunteers (CHVs) and Mother-to-Mother Support Group as per our power analyses tools (See Supplementary File 1). In each category of the FGD groups, attempts were made to have a homogenous group of participants. For quality control purposes, the data enumerators were trained on the procedures and ethical issues related to the data collection and the instruments were pre-tested prior to use. During the FGD, one investigator led the tape-recorded discussions (DOO). The collection of data was performed under the supervision of the investigators (COO, CO, DOO, SG). In each case, Kipsigis (local language), Kiswahili or in exceptional cases, English, was used as medium of communication.

#### Questionnaire-interview method

Initially, a questionnaire was administered to each respondent by an enumerator for a period of about 45 min to collect data on critical items such as Collection and use of Iron and Folic Acid Supplementation (IFAS), Regular nutrition assessment, Practice of quality of diet, Use of RUTS/RUSF (Ready-to-use Therapeutic Food and Supplementary Food/ Ready-to-use Supplementary Food, Vitamin A supplementation for the child, Use of ITNs, Regular visit for Nutrition education and counselling and overall adherence to utilization (See Supplementary File 2). These were treated generally as proxy quality indicators. Utilization pattern associated with nutrition services was assessed in such a way that participants who scored 4 or more items against a scoring rating between 4 and 5 were labelled ‘good utilizers’ while those who scored between 1 and 3 were labelled ‘bad utilizers’. Good utilizers were assumed to have high chances of accessing quality of nutrition services.

#### Focus group discussions (FGDs) and power mapping analyses

As part of the power mapping analyses, five (5) focus groups targeting Community Health Workers, adolescent fathers and spouses, adolescents pregnant and lactating, adolescent mothers, and Mother-to-Mother Support Group were conducted to examine the power dynamics in the families and communities and their impact on the pregnant and lactating adolescent girls’ access to nutrition services in Trans-Mara East Sub-County, Narok County (Supplementary File 1). Each group consisted of 8 participants. The analysis was guided by the investigators to ensure validity of data. The process was participatory in nature i.e. we allowed study participants to take part as actively as possible and get involved fully in the discussion activities.

### Statistical analyses

*Quantitative data analysis* adopted use of descriptive and inferential statistics. Descriptive statistics was used to characterize different frequencies. Z-test for single proportions was used to test for significant difference between the actual frequencies and expected frequency. Expected frequency was set at 50% for dichotomized data and 100/n percent for data that had more than two options. Principal Axis Factoring was used to establish the access pattern as well as generating *Batt*-scores for further modeling especially for indicators that were fitted into access and utilization models to determine cause and effect. *Qualitative Data Analysis* on the other hand adopted the use of *Framework analysis* for both in-depth interviews and Focus Group Discussions. In the *Framework analysis*, comparisons with single expected frequencies were made as a probability of the possible outcomes for each variable addressed. For dichotomous data, assumption was made at 50% while variables that had more than two categories were assigned 100/n expected frequencies. For the *Power Analysis*, the stakeholders involved were represented by board game figures that are characterized through “range-of-action-cards” and put on wooden “power towers” to show their power in influencing access and utilization of nutritional services, and the participants were allowed to demonstrate whether the power of influence was visible, hidden or invisible. The result was a three-dimensional sketch that provided quantitative data and guided the qualitative discussion about reasons for and effects of the power of the different stakeholders. Relationship between individual power dynamics and overall utilization of nutrition services were determined using binary regression analyses. For the regression analyses, the factor load of the variable “Power dynamics” was categorical in order to conduct a regression with a binary outcome to estimate an odd ratio. In all analyses, *P* ≤ 0.05 was considered statistically significant.

## Results

### Power dynamics

#### Individual power dynamics

Individual-based power dynamics focused on the adolescents (*N* = 292), where power factors were examined within three latent domains: self-esteem, social position and gender dynamics.

#### Self-esteem

Table 1 present data on self-esteem as assessed based on eight indicators. The items were subjected to principal axis factoring and the results isolated two distinct factors loading cumulatively and accounting for 38.8% of total variance on self-esteem. Factor 1 loaded five indicators made up of ability to resolve problems (cum = 0.632), always pushing to seek service (cum = 0.633), always getting some way to deal with problems (cum = 0.633), ability to overcome spouse/parent/guardian decision (cum = 0.598) and can act to ensure healthy eating during hard times (cum = 0.446). This category depicted *“intrinsic capability”* as enhancers to access to nutrition services and accounted for 24.5% of total variance in self-esteem.

**Table 1:** Self-Esteem power matrix in the context of nutrition and health status of adolescent who are lactating and pregnant

Indicators of Self-Esteem	Categories of Self-Esteem	
	1	2
Able to resolve nutrition and dietary problems on my own	0.632	
Dependent on significant others to resolve nutrition and dietetics problems		0.560
Always push and get what she wants when seeking nutrition services from a health facility and somebody opposes her	0.633	
Always get some way to deal with health and nutrition problems that confronts me.	0.633	
Can overcome spouse/guardian/parents’ contrary decision to seek nutrition and health services	0.598	
Can act to improve nutrition status through healthy eating during hard times	0.446	
Decision to seek health and nutrition services is always directed by significant others (e.g. parents, spouse, guardian, siblings)		0.694
Always find it difficult to deal with health and nutrition problems that confronts me.		0.651
**Overall Variance**	**24.5**	**14.3**

Table present data on self-esteem as assessed based on eight indicators. The items were subjected to principal axis factoring and the results isolated two distinct factors loading cumulatively and accounting for 38.8% of total variance on self-esteem. *Factor 1* intrinsic capability and *Factor 2* extrinsic capability. *Cum* 0.4 is significant

The second factor loaded three items including dependency on significant others (cum = 0.560), seeking direction from significant others (cum = 0.694) and finding difficulty to deal with nutrition problems (cum = 0.651). This category of factors was labelled *“extrinsic dependency”* self-esteem. This category depicted negative attribute as barriers to access to nutrition services and accounted for 14.3%. Considering the two factors, it appears that enhancers outweighed the barriers in the context of access and utilization of nutritional services by these adolescents (Table 1).

In an FGD to triangulate the above information, mothers, CHVs and parents of adolescents also expressed their opinion on aspects of self-esteem. Critical voices from the adolescents who were mothers reflected inability to resolve their nutrition and dietary problems on their own, thus depicting a dependency factor. Parents and CHVs also confirmed dependency factor through voice commonly sounding as *“high risk”*. This was tied to additional problems from men who offer to assist the girls in exchange of sex. This scenario pushes parents to continue supporting the adolescents on the matter of nutrition and healthcare. These are depicted from the following statements from the FGDs:

*R3-they don’t, so us as CHVs, we usually explain to parents so that no other person will assist … .*

*R8-they don’t resolve, in presence of their parents, they do so ….*

*R6-we are not able because we are still young even our energy is not sufficient…*

*R7- I am not able because I still depend on my parents...*

*R1-unmarried is unable to resolve problems by her own. If in case she is not assisted mafisi* 1*will come and assist hence bringing in more problems….*

*R2- I don’t think they do because age 10–19 is still young and they depend on parents. Tegemeo ni mzazi.* 2*Unless she has a small garden.*

#### Social position

Table 2 presents data on perceived social position for the target group using 15 indicators. Based on principal axis factoring, the 15 indicators loaded up to four factors accounting for 46.6% of total variance of social position.

**Table 2:** Social Position power matrix in the context of nutrition and health status of adolescent who are lactating and pregnant

Social Position Indicators	Social Position Categories			
	1	2	3	4
Adolescent receives respect and care from spouse respects about health and nutrition status	0.445			
Adolescent receives respect and care from people she lives with on health and nutritional status	0.546			
Adolescent receives respect and care from in community about my health and nutrition status	0.604			
Adolescent receives respect and care from people in church about health and nutrition status	0.466			
Adolescent accepts that father value her opinion				0.601
Adolescent accepts that guardian value her opinion			0.648	
Adolescent accepts that spouse still show willingness to support my education despite pregnancy/lactation status		0.647		
Adolescent accepts that her guardian takes full responsibilities in educating her regardless of her status			0.621	
Adolescent accepts that father takes full responsibilities in educating her regardless of my status		0.708		
Adolescent accepts that mother takes full responsibilities in educating her regardless of my status.		0.720		0.445
Adolescent accepts that mother value my opinion				0.724
Adolescent accepts that members of extended families value my opinion			0.443	
Adolescent accepts that peers within the community demonstrate socio-support whenever she is in need.	0.478			
Adolescent accepts that she can allocate money to seek health and nutrition services without permission from parents/spouse/guardian/siblings	0.550			
Adolescent accepts that she is able to decide on which areas of her needs require resources to be allocated.	0.642			
**Overall Variance Overall Variance**	**15.4**	**13.1**	**9.1**	**9.0**

Table presents data on perceived social position for the target group using 15 indicators. Based on principal axis factoring, the 15 indicators loaded up to four factors accounting for 46.6% of total variance of social position. *Cum* 0.4 is significant

The first factor which accounted for 15.4% isolated critical indicators which depicted care, respect and social support from key stakeholders mainly spouse (cum = 0.445), family members (cum = 0.546), community (cum = 0.604), church (cum = 0.466) and peers (cum = 0.478) and capability to make own decisions (mainly by ability to allocate resources; cum = 0.550) and resource need identification (cum = 0.642). This category of social support was labelled, “Care, respect, social support and decision-making capability.” However, adolescents who were mothers appeared to voice during focus group discussions that some of them have limited freedom on use of resources thus diverging from the quantitative outcome. In some instances, they get allocation of small pieces of land to plant their crops to meet their needs. However, parents and CHVs voiced “*limited freedom*” as majority are still under guidance. Adolescents voiced out *“selective approaches in resource allocation”* as well as *“mistreatment”* especially where household family size appears big and every member need attention.

*R3-No, they don’t have any resources unless the parent provides….*

*R1-This teenage girl at times have the small garden which can assist her to meet her needs. Also, at times she might have chicken….*

*R6- I see that they lack resources because they are still under parent’s protection still….*

*R3- Yes, I was given freedom of time but other resources such as land we are usually denied especially us as girls…*

*R1- they can get freedom after consulting from the parent. But if she does not ask, she will not have that freedom. Also, if child has a lot of freedom she might misbehave ….*

*R2- Yes, but freedom with restrictions. I should know where she is and also, we should consult with each other all the time...*

The second factor which accounted for 13.1% of variance on social support loaded three sub-indicators which focused on education support from spouse (cum = 0.647), father (cum = 0.708) and mother (cum = 0.720). This shows that mother and father were more concerned about adolescents’ education as compared to their spouse, though the spouse concern was equally reasonable thus category of social position was labelled “family-specific education support”. Parents, CHVs and adolescent lactating somehow agreed on a common voice on parental commitment to education support for adolescents who are pregnant and lactating. This demonstrates parental commitment to education of girls despite pregnancy status. However, multiple pregnancies are not entertained and would lead to neglect.

*R11-Nowadays, parents have known importance of education since it will later reduce poverty cases…*

*R9-I deliver and they say I finish six months then I go back to school…*

*R8- I was told to go to school immediately three months after delivery…*

*R1-Me after I delivered, I was supported fully since no other child were educated at home …*

*R4- Yes, they really support…*

*R5-some parents are strict such that if you give birth consecutively they cannot support you to go back to school*

The third factor loaded three sub-indicators focusing on guardian and extended family relationships with the adolescents. Guardian (cum = 0.6548) and extended families’ (cum = 0.443) value for adolescent’s opinion coupled with full responsibility taken by guardian (cum = 0.621) emerged as a third level social position accounting for 9.1% of social support. This category was labelled “guardian/extended family concern”.

The fourth factor focused on four sub-indicators totaling 9.0%. These included opinion from father (cum = 0.601), mother (cum = 0.724) and mother’s education responsibility (cum = 0.445). This factor was labelled parental value for opinion in the context of adolescents’ nutrition and health (Table 2).

#### Gender dynamics

Table 3 presents data on gender dynamics indicators loaded into two factors with distinct thematic focus accounting for 47.8% of overall gender dynamics. The first factor identified respect for adolescent (cum = 0.522), community push for equal treatment (cum = 0.580), equal treatment on youth leadership (cum = 0.687), equal opportunity to pursue education as boys (cum = 0.742) and equal opportunity for resource allocation (cum = 0.497). This category of gender dynamics was labeled “gender equity” as depicted by strong perception of the adolescents.

**Table 3:** Gender dynamics power matrix in the context of nutrition and health status of adolescent who are lactating and pregnant

Gender dynamic indicators	Factor	
	1	2
Adolescent accepts that members of this community have respect to pregnant/lactating adolescents	0.522	
Adolescent accepts that members of this community treat girls and boys equally	0.580	
Adolescent accepts that both pregnant/lactating adolescent girls and boys of same age group are given equal opportunity in youth leadership	0.687	
Adolescent accepts that both boys and pregnant/lactating adolescent girls of the same age group receive equal opportunity to pursue education	0.742	
Adolescent accepts that both boys and pregnant/lactating adolescent girls receive equal opportunity in resource allocation	0.497	
Adolescent accepts that cases of gender-based violence against girls who are pregnant or lactating are common in this community.		0.517
Adolescent accepts that pregnant/lactating adolescent girls sometimes receive psychological, emotional and verbal abuse when they visit health facilities		0.557
Overall Variance	**27.1**	**10.7**

Table presents data on gender dynamics indicators loaded into two factors with distinct thematic focus accounting for 47.8% of overall gender dynamics. *Factor 1* intrinsic capability and *Factor 2* extrinsic capability. *Cum* 0.4 is significant

The second factor loaded on two sub-indicators namely gender-based violence (cum = 0.517) and psychological, emotional and verbal abuse (cum = 0.557). This category of gender dynamics was labeled “physical and emotional violence” in relation to seeking nutrition and health care. This accounted for 10.7% of gender perspective of adolescent health (Table 3).

### Individual power dynamics and utilization of nutrition services

Table 4 presents logistic regression analyses results on how individual power dynamics influence overall utilization of nutrition services. The intrinsic capability was more likely to decrease awareness by half (OR = 0.52, 95% CI = 0.4–0.7, *P* < 0.0001). Somehow, extrinsic dependency was likely to increase utilization by 1.2 times (OR = 1.2, 95% CI = 1.0–1.5, *P* = 0.055). Again, within the social support domain, education support was also likely to decrease overall utilization by 0.8 times (OR = 0.8, 95% CI = 0.6–0.96, *P* = 0.021). Other isolated factors within the social support had no significant influence on utilization. Within the gender dynamics, gender equity was more likely to increase utilization by 1.4 times (OR = 1.4, 95% CI = 1.1–1.8, *P* = 0.007) (Table 4).

**Table 4:** Relationship between individual power dynamics and overall utilization of nutrition services

Power Dynamics	OR	95% CI	*P*-value
**Self Esteem**			
Intrinsic capability ***	0.52	0.4–0.7	**< 0.0001**
Extrinsic dependency *	1.2	1.0–1.6	**0.055**
**Social Support**			
Care, respect, social support and decision-making capability	1.1	0.8–1.5	0.621
Education support*	0.8	0.6–0.96	**0.021**
Guardian/extended family concern	0.9	0.7–1.0	0.126
Parental value for opinion	1.0	0.8–1.2	0.604
**Gender dynamics**			
Gender equity **	1.4	1.1–1.8	**0.007**
Physical and emotional violence	0.9	0.7–1.1	0.283

Table presents binary regression analyses results on how individual power dynamics influence overall utilization of nutrition services. * *p* < 0.05; ** *p* < 0.01; *** *p* < 0.001 based on z-test effect size; *OR* odds ratio; refers to the likelihood of having the listed power dynamics i.e. self-esteem, social support and gender dynamics, *CI* confidence interval

Assessment of additional social barriers to utilization of nutrition services from CHVs, mothers and parents generally surrounded voices of *“shame”*, *“complacence”*, *“fear”*, *“verbal abuse”* and *“limited knowledge”* on new approaches in medicine. Furthermore, these issues surrounded social aspects of healthcare which isolated only nutrition-sensitive matters in the adolescent life as captured below.

*R10-they feel ashamed because they are still young like the case I experienced with class six girls last year ….*

*R6-whenever she sees she is well she sees no need of visiting hospital….*

*R9-the problem of one immunization is that they get one dose and assume that is enough ….*

*R5-when you are married you find you are so much committed and nobody will be left to take care of home … …*

*R1-I was fearing because I thought my baby’s weight was too low ….*

*R7-mostly it is fear, but if she is free to say parent can willingly take her ….*

*R2-parents are at times stressing the lactating girls and abuse saying that they are useless. Big challenge is parent because even they cannot cloth this girl or even give her information…*

*R8-parents lack information of current technology and due to that they prefer using herbal medicines*

### Stakeholder power dynamics

Table 5 presents data on stakeholder power dynamics as they revolved around seven stakeholder domains identified in a participatory manner through power mapping analysis where adolescents, parents and CHVs were involved. Two critical thematic issues that were analyzed included access and utilization of nutrition services by adolescents who were pregnant or lactating.

**Table 5:** Stakeholder Power Identification

Stakehoder Power	Access to nutrition services			Utilization of Services and Pieces of advise		
	Visible power	Hidden Power	Invisible Power	Visible power	Hidden Power	Invisible Power
Adolescent		Adolescent				Adolescent
Family	Parents		CHVs			
Community		Parents CHVs	Parents		Adolescent CHVs	
Partners (e.g. NGOs)	Adolescent Parents	CHVs		Parents	CHVs	
Health perssonel	Adolescents CHVs Parents			Adolescents CHVs Parents		
Political	CHVs		Parents			
Government	CHVs	Adolescent		Parents		CHVs

Table presents stakeholder power dynamics as they revolved around seven stakeholder domains identified in a participatory manner through power mapping analysis where adolescents, parents and CHVs were involved. Two critical thematic issues analyzed included access and utilization of nutrition services by adolescents who were pregnant or lactating

Adolescent as primary stakeholders had power to enhance or bar access and utilization of nutrition services. As enhancers, adolescent power was characterized by knowledge, education, wealth and awareness about support factors leading to accessing nutrition services. These were identified as medium rating falling within the upper 3rd of 100th percentiles. On the other hand, adolescent identified poverty, ignorance, laziness, stigma, shyness and lack of awareness on nutrition matters as critical barriers. However, this fell within the lower 3rd of 100th percentiles displaying a scenario where enhancers outweighed barriers. In the power matrix, adolescent power was identified as a “hidden power” on matters of access to nutrition services and “invisible power” on matters of utilization of nutrition services.

Family as a stakeholder power domain symbolized by father or spouse, mother, grandmother and siblings also play a key role in access and utilization of nutrition services. Father’s push towards access was characterized by support to education, provision of basic needs and health security which accounted for power that fell within the middle 3rd of 100th percentiles. *Fathers* also acted as barriers to access and utilization of nutrition services by restricting freedom, provision of limited support to education, limited monetary support and limited support towards health care of adolescents who are pregnant of lactating. *Fathers* engaged in unhealthy talk and abandonment of the adolescents. This also fell within upper 3rd of 100th percentiles. This implies that *fathers* had more power to prevent access and utilization of services. *Mothers*, on the other hand, being critical significant in access and utilization of nutrition services contributed towards access by providing food, social security protection, nutrition counseling, education and basic needs. However, this only fell within the lower 3rd of 100th percentiles. *Mothers* could also act as barriers by limiting nutrition counseling and education, restricting food, supporting abortion and giving improper advice and experiencing poverty. This accounted for 3rd of 100th percentiles. It appeared that mothers have less power to ensure access and utilization of nutrition services by the adolescents. *Grandmothers* were also mapped out as key stakeholders with power to enhance access and utilization of nutrition services. *Grandmother* was identified to be critical on advising and counseling adolescents with power that fell in the upper 3rd of 100th percentiles. On equal measure, grandmothers could demotivate the adolescents from attending clinic and delivering at a health center. This was also rated within the 3rd of 100th percentiles. This outcome defined a *neutral power* from the grandmothers. *Siblings* as part of the family domain had power to influence adolescents’ access and utilization of nutrition services through provision of social support and security. This power was rated within the 3rd of 100th percentiles. *Siblings* at times would act as barrier by denying adolescents who are pregnant and lactating material and social support. *Siblings* seldom engaged in counseling processes for the adolescent thus rating this barrier power within the middle 3rd of 100th percentiles. Further analysis to review the nature of power revealed that family power was perceived as “visible power” by parents and “invisible power” by the CHVs.

Community structures also exhibited power as a push or barrier to access and utilization of services. Village elders, CHVs, midwives and pastors have power to influence access and utilization, which rated within the upper 3rd of 100th percentiles. This power was characterized by provision of counseling and advice on clinical nutrition, screening and use of FP, IFAS, assistance of home delivery and spiritual support. On the contrary, barriers were attached to village elders on account of limited counseling, limited material support and freedom. Some CHVs were identified as frequent in concealing pregnancy status of adolescents while pastors were blamed for failure to offer spiritual support. The barriers somehow fell within the upper 3rd of 100th percentiles. The position of the community structure in the stakeholders’ matrix, also display neutral effect on access to nutrition services. A review of the nature of community power revealed a hidden power associated with the community systems.

Partners as a stakeholder characterized by FBOs and NGOs demonstrated a bigger influence through support to nutrition education, supply of basic items and supporting capacity building of CHVs. However, these provisions were noted to be inadequate. Adolescents and parents identified partners as “visible power” for access to nutrition services. The CHVs on the other hand identified partners as “hidden power”.

Health personnel were mapped out as key stakeholders in the provision of food supplements, nutrition education and counseling, which was mainly provided for by nutritionists. Nurses and medical doctors provided appropriate treatment, social advice and medicine; clinical officers were identified as key providers of immunization while public health officers assisted on training of CHVs on disease surveillance. Higher rating was attached to health personnel as enhancers to nutrition service delivery. However, barriers associated with health personnel included failure to deliver adequate information, denying adolescents treatments, lack of proper medicine and advice, absenteeism (mainly associated with clinical officer) and inadequate training for CHVs. However, these barriers were rated low and fell within the lower 3rd of 100th percentiles, giving more weight to enhancers. Further analysis revealed that health personnel were unanimously identified as “visible power” by adolescents, parents and CHVs.

The political stakeholders were equally mapped out to be critical in nutrition service delivery. Political figures mainly MCAs who provide monetary support and food which MPs provide in the form of CDF to build health facilities. Their rating as enhancers attached a bigger weight. This category of stakeholder also acts as barriers when they fail to provide physical infrastructure or education support. The MPs were associated with corruption leading to bad roads which then lead to failure of access to nutrition services.

Government was mapped out as key power influencer. As facilitators of access to nutrition services, both national and county governments provided basic social needs including education and health support. They were identified as major financers of health with a greater weight attached to their power. On the contrary, they acted as major barriers specifically on their support on delayed supply of medicine and poor roads within Trans-Mara East Sub-County. Barriers were rated highly by the participants (Table 5). Parents and CHVs identified government as a “visible power” while adolescents view them as “hidden power”. Perhaps, the adolescent may not be aware about the government’s role in the provision and support of nutrition services.

## Discussion

### Power dynamics and utilization of nutrition services

#### Individual dynamics

This study assessed self-esteem in the context of utilization of nutrition services where *“Intrinsic Capacity”* was isolated to be heavier than the *“Extrinsic Dependency”*. This implies that many adolescents were making attempts and were struggling on their own to meet their nutritional requirements. However, attempts to establish how these two categories of self-esteem components appear to demonstrate *“extrinsic dependency”* having the potential to improve overall utilization while *“intrinsic capacity”* demonstrated potential to reduce utilization. With this kind of outcome, it is clear that adolescents depend on significant other to perform better than those who push to solve their nutrition problems. This latter case could be attributed to social struggle with limited resources and social security which consequently suppress their access to nutrition advice and services.

In the context of social support, and in particular, education support, the study revealed that *“education support”* given to adolescents reduced level of utilization of nutrition services. Somehow, that was an unexpected outcome from the current study, however, it is apparent that adolescents given a second chance, would focus more on their education than caring for their nutrition needs to please their parents or guardian. Several reasons could be attributed to our observations. It has been previously established that an effective way to reach adolescents and provide nutritional knowledge is by the promotion of behaviour-change communication (BCC) between school-going and out-of-school adolescent girls in the community [14]. As much as we could not establish how many of the adolescents (whether pregnant or lactating) have gone through BCC, community-based BCC may help create an enabling environment for girls in the household and community as it focus on healthy lifestyles as entry-points with information on more sensitive topics, such as human sexuality, sexually transmitted disease, and substance abuse, in addition to nutrition-based services. Periodic assessment of knowledge and practice of adolescent girls may guide the discussion issues in accordance with their need. Some operations research may be needed to develop communication materials and additionally provide critical information about how to sensitize policy-makers, service providers, and teachers to work together and to increase participation in community-based adolescent girls’ forums. Undoubtedly, these observation could also further shed some light on shifting intervention site from health centers to schools to respond to the adolescents who find themselves in such dilemma.

Gender equity also demonstrated potential to increase utilization of nutrition services. Gender equity demonstrated that parents, community and guardian provide a fair treatment to both boys and girls regardless of the status of the girls (whether pregnant or lactating). This brings comfort and more focused concerns for the adolescent nutritional health from the adolescent themselves and their parents, guardian or community. It is on the basis of this confidence that adolescents find it easy to freely seek good nutrition and health care. Additionally, parents also find it easy to ensure adolescent’s nutrition service is properly met.

### Stakeholder power matrix

The study attempts to map out key stakeholders with visible, hidden and invisible powers that would influence adolescents’ access and utilization of services. Six categories of stakeholders included family, community, partners, health personnel, political and the government.

It appeared that health personnel had the biggest weight as attached by participants in the power mapping process. Health personnel had observable visible power to influence access and utilization of nutrition services thus demonstrating a direct link between health care and the adolescents. Family, partners, political and government also had visible power of influence while the community seemed to demonstrate hidden and invisible power.

Stakeholder matrix is a factor that fits squarely in strengthening systems. Health personnel are properly trained to handle technical aspects of healthcare delivery. Partners in most cases, set up interventions and provide resources that complement government existing systems. The political and government systems are anchored on the political and economic theory with responsibility to protect the vulnerable in a society [15].

The responsibility of ensuring access and utilization in the context of this study was also identified to depend on family systems where adolescents themselves, parents, siblings and guardians had a direct role to play in access and utilization of nutrition services. Our study made attempts to review each stakeholder in a mapping process where adolescents, pregnant and lactating, parents and CHVs participated. The study identified major gaps on stakeholder networking for a common goal as each stakeholder still operated on its own with a clear limited integration. Management of key stakeholders in projects is widely supported as an important aspect in service delivery [16, 17] as stakeholder have the power to influence service positively or negatively [18].

In the current study, the adolescents also identified key stakeholders. These stakeholders needed to be organized in an integrated network density which is a basic measure characterized as a “whole network concept” [19], and which has more power than each pillar in the network. Identification of central actors in the stakeholders’ matrix would be useful in determining appropriate locus for service delivery for the adolescents who are pregnant and lactating as power allocation would be focused on the actor as coordination point for effective monitoring of performance. The power visibility for the stakeholders was still a hidden issue on matters related to the adolescents who are pregnant and lactating. However, it was pointed out that enhanced stakeholder engagement have a higher potential of increasing access and utilization of services through dialogue.

Finally, it is worth pointing out that observational and questionnaire-based study have some limitations. For instance, the results from the current study is used to generalize conclusions on the whole of lactating and pregnant adolescents in Narok County as much as the study is focusing on Trans Mara East Sub-County. However, the tools designed and the sample size targeted made attempt to capture the exact issues revolving around access to nutritional services by the lactating and pregnant adolescents in Trans Mara East Sub-County, Narok County, Kenya. Furthermore, individual surveys are not good at following trends in real-time or over short periods of time. Since surveys collect data at a single point in time, it is difficult to measure changes in the population unless two or more surveys are done at different points in time. Such repetition is often expensive and time-consuming, making frequent periodic surveys impractical. In addition, a single cross-sectional survey cannot disentangle the different contributions of each of factors to lead us into a cause and effect relationship.

## Conclusion

The study assessed individual power as a factor in access to adolescent nutrition services. The results revealed that adolescents who draw their support from significant others were more likely to utilize nutrition services as compared to those who attempted to use their individual initiative to seek such services. This implies that most adolescents still depend on parents or guardians for support and any attempt to allow them to take care of their nutrition needs is high risk. Unexpectedly, further education support to adolescents who are pregnant turned out to be a barrier to access nutrition services. In the stakeholder domain and their power of influence in adolescents’ health, health personnel appeared to have the most influential powers in ensuring adolescents access nutrition services and would be the most important actors in the stakeholder matrix. Other actors identified as useful powers were parents, political figures and governments. Stakeholder engagement was identified to have higher potential of increasing access and utilization of services through dialogue. There is therefore need to increase access to adolescents within the community set up through considerable scope for making better use of multiple avenues to reach adolescents, including school-based, health system-based and community-based approaches and even marriage registries. There is also need to heighten engagement in the stakeholder network (family, community, partners, health personnel, political and government) when planning community conversations, to ensure a multi-stakeholder approaches in meeting the nutrition needs of adolescents.

## Supplementary information

**Additional file 1.** These are the dataset supporting the conclusions of this article which is provided as Supplementary File 1.

**Additional file 2.**

**Additional file 3.**

## Data Availability

The dataset supporting the conclusions of this article is included within the article as Supplementary File 2.

## Abbreviations

BCC: Behavior-change communication
CDF: Constituency development fund
CHV: Community health volunteers
FBOs: Faith based organizations
FGDs: Focus group discussions (FGDs)
FP: Family planning
HIV: Human immunodeficiency virus
IFAS: Iron and folic acid supplementation
KNDI: Kenya Nutritionists and Dieticians Institute
MCAs: Member of County Assembly
MPs: Member of parliament
NGOs: Non-Governmental Organizations
RUTS: Ready-to-use therapeutic food and supplementary food
RUSF: Ready-to-use supplementary food

## Footnotes

1 *Mafisi* means hyenas and is used to refer to men who are ready to have sex with girls in exchange for any assistance.

2 *Tegemeo ni mzazi* means depend on parents. It is used to imply that they depend entirely on parents to meet their needs.
