# Supplementary material for: Power dynamics as a determinant of access and utilization of nutrition services by pregnant and lactating adolescent girls in Trans-Mara East Sub-County, Narok County, Kenya
Source: BMC Public Health. 2020 Apr 19;20:537. doi: 10.1186/s12889-020-08690-w (PMC7168838; doi:10.1186/s12889-020-08690-w)
Supplement: Supplementary file 1 — Additional file 1. These are the dataset supporting the conclusions of this article which is provided as Supplementary File 1. [file 12889_2020_8690_MOESM1_ESM.docx]

## **Supplementary File I: Adolescent Questionnaire**

|  | **FORMATIVE RESEARCH FOR THE PROJECT**  ‘**IMPROVING ADOLESCENT NUTRITION PROJECT IN NAROK COUNTY, KENYA’** | |
| --- | --- | --- |
|  | **ADOLESCENT QUESTIONNIAIRE** |  |
| Q | **Questions** | **Options** |
| 1.0 | IDENTIFICATION |  |
| 1.1 | Name of the Data Collector |  |
| 1.2 | Survey date (dd/mm/yy) |  |
| 1.3 | County |  |
| 1.4 | Sub-County |  |
| 1.5 | Ward | Olomasani |
|  |  | Mogondo |
|  |  | Kapsasian |
|  |  | Likerin |
| 1.6 | Cell phone number of respondents |  |
| 1.7 | Gender of household head | 1. Male |
|  |  | 1. Female |
| 2.0 | **RESPONDENT’S SOCIO- ECONOMIC AND DEMOGRAPHICS STATUS** | |
| 2.1 | Respondent’s Study ID |  |
| 2.2 | Adolescent status | 1. Pregnant |
|  |  | 2. Lactating |
| 2.3 | Marital status of the adolescent | 1. Married |
|  |  | 2. Single living a lone |
|  |  | 3. Widowed |
|  |  | 4. Separated |
|  |  | 5. Divorced. |
|  |  | 1. Single living with parent/guardian |
| 2.3 | The adolescent’s spouse Study ID | _____________________________ |
| 2.4 | The adolescent’s parent/guardian Study ID | _____________________________ |
| 2.5 | Age of the adolescent | _____________________________ |
| 2.6 | Total number of people living in the household where adolescent reside | _____________________________ |
| 2.7 | Last level of education attained | 1. University/tertiary on going |
|  |  | 1. Secondary completed |
|  |  | 1. Secondary ongoing |
|  |  | 1. Primary completed |
|  |  | 1. Primary on going |
|  |  | 1. Stopped going to school |
| 2.8 | What is your religion currently? | 1. Christian |
|  |  | 1. Muslim |
|  |  | 1. Traditional |
|  |  | 1. Hindu |
|  |  | 1. Other Specify ___________________ |
| 2.9 | What are your sources of food? …………………………… |  |
|  |  |  |
|  | 1. Own agricultural production ……………………………. | 1. Yes |
|  |  | 2. No |
|  | 2. Purchase from market …………………………………… | 1. Yes |
|  |  | 2. No |
|  | 3. Food aid…………………………………………………… | 1. Yes |
|  |  | 2. No |
|  | 4. Donation from neighbors and/or friends…………………... | 1. Yes |
|  |  | 2. No |
|  | 5. Church donation …………………………………………… | 1. Yes |
|  |  | 2. No |
|  | 6. Dependent on parent or guardians …………………... | 1. Yes |
|  |  | 2. No |
| 2.10 | What is your **main** current source of income? |  |
|  |  | 1.        Agriculture (crop growing) |
|  |  | 2.        Livestock herding |
|  |  | 3.        Casual Labor |
|  |  | 4.        Self-employed |
|  |  | 5.        Skilled labor |
|  |  | 7.        Salaried employment |
|  |  | 8.        Petty trade |
|  |  | 10.     Charcoal burning |
| 2.11 | What is your **average monthly** income? . | ____________________Ksh |
| 3.0 | **INDICATORS OF ACCESS TO NUTRITIONAL ADVICE AND SERVICES**  (Information booklet to be provided to enumerators during training) | |
| 3.1 | Have you received any nutrition advice in the past three months? | 1. Yes |
|  |  | 2. No (If no skip 3.3) |
| 3.2 | Have you received any nutrition service in the past three months? | 1. Yes |
|  |  | 2. No |
| 3.3 | What domain of nutrition advice did you receive in the past three months? (if yes question 3.1) |  |
|  | 1. Advice on Healthy diet/diet diversity | 1. Yes |
|  |  | 2. No |
|  | 1. Advice on exclusive breastfeeding | 1. Yes |
|  |  | 2. No |
|  | 1. Advice on nutrient supplementation | 1. Yes |
|  |  | 2. No |
|  | 1. Advice on food fortification and blending | 1. Yes |
|  |  | 2. No |
|  | 1. Advice on appropriate complementary feeding (for lactating mothers only) | 1. Yes |
|  |  | 2. No |
|  | 6. Any other _____________________________________ | 1. Yes |
|  |  | 2. No |
|  |  | 1. Yes |
|  |  | 2. No |
| 3.4 | What domain of nutrition service did you receive in the past three months? (if yes question 3.2) |  |
|  | 1. Provision and collection of IFAS | 1. Yes |
|  |  | 2. No |
|  | 1. Nutrition education and counseling | 1. Yes |
|  |  | 2. No |
|  | 1. Deworming | 1. Yes |
|  |  | 2. No |
|  | 1. Vitamin A supplementation for the child | 1. Yes |
|  |  | 2. No |
|  | 1. Sexual and reproductive health sensitive to nutrition e.g. family planning | 1. Yes |
|  |  | 2. No |
|  | 1. Basic environmental hygiene, and disease prevention e.g. provision of ITNs | 1. Yes |
|  |  | 2. No |
|  | 1. Basic personal hygiene | 1. Yes |
|  |  | 2. No |
|  | 1. Regular nutrition assessment both at antenatal and postnatal | 1. Yes |
|  |  | 2. No |
|  | 1. Child growth monitoring at postnatal care | 1. Yes |
|  |  | 2. No |
|  | 1. Nutrition referral for critical malnutrition episodes | 1. Yes |
|  |  | 2. No |
|  | 1. Nutrition support e.g. mother to mother support | 1. Yes |
|  |  | 2. No |
|  | 1. Nutrition supplements e.g. ready to use therapeutic/Supplementary foods RUTS/RUSF | 1. Yes |
|  |  | 2. No |
|  | 1. Regular follow-ups on utilization of services e.g. through community strategy programmes | 1. Yes |
|  |  | 2. No |
|  | 1. Lactation management and processes e.g. normally done using lactation charts pathways | 1. Yes |
|  |  | 2. No |
| 3.5 | Who provided pieces of advice or services mentioned 3.3 and 3.4? |  |
|  | 1. Nutritionists | 1. Yes |
|  |  | 2. No |
|  | 1. Nurse | 1. Yes |
|  |  | 2. No |
|  | 1. Physician/doctor | 1. Yes |
|  |  | 2. No |
|  | 1. CHVs | 1. Yes |
|  |  | 2. No |
|  | 1. Community Development Social Worker | 1. Yes |
|  |  | 2. No |
|  | 1. Pharmacists | 1. Yes |
|  |  | 2. No |
|  | 1. Other (Specify)___________________________________ | 1. Yes |
|  |  | 2. No |
|  |  | 1. Yes |
|  |  | 2. No |
| 3.5 | Where were the pieces of advice/services provided? |  |
|  | 1. Public dispensaries | 1. Yes |
|  |  | 2. No |
|  | 1. Private clinic | 1. Yes |
|  |  | 2. No |
|  | 1. Private hospital | 1. Yes |
|  |  | 2. No |
|  | 1. Public hospital | 1. Yes |
|  |  | 2. No |
|  | 1. Public Health Centre | 1. Yes |
|  |  | 2. No |
|  | 1. CBO and NGO health project | 1. Yes |
|  |  | 2. No |
|  | 1. FBO project | 1. Yes |
|  |  | 2. No |
|  | 1. Public health clinics | 1. Yes |
|  |  | 2. No |
|  | 1. At School | 1. Yes |
|  |  | 2. No |
| 3.6 | How far in the source of advice/service from your residence? |  |
|  | 1. Less than 1 km | 1. Yes |
|  |  | 2. No |
|  | 1. 1-3 km | 1. Yes |
|  |  | 2. No |
|  | 1. Above 3 but less than 5km | 1. Yes |
|  |  | 2. No |
|  | 1. 5-10km | 1. Yes |
|  |  | 2. No |
|  | 1. Above 10km | 1. Yes |
|  |  | 2. No |
| 3.7 | How were the nutrition advise information conveyed to you in the past three months? |  |
|  | 1. IEC materials e.g. brochures, leaflets etc. | 1. Yes |
|  |  | 2. No |
|  | 1. Bulk SMS | 1. Yes |
|  |  | 2. No |
|  | 1. Internet links referrals | 1. Yes |
|  |  | 2. No |
|  | 1. Face to face | 1. Yes |
|  |  | 2. No |
|  | 1. Video clips | 1. Yes |
|  |  | 2. No |
|  | 1. Social media e.g. WhatsApp and Facebook pages | 1. Yes |
|  |  | 2. No |
| 3.8 | Rate your level of adherence to utilization of the following critical nutrition and health services | *Choose the most appropriate choice by circling the correct option.*  1=Strongly disagree, 2=Disagree, 3=neither disagree/agree, 4=agree, 5=Strongly agree |
|  | 1. Collection and use of IFAS | 1 2 3 4 5 |
|  | 1. Regular nutrition assessment | 1 2 3 4 5 |
|  | 1. Practice of quality of diet | 1 2 3 4 5 |
|  | 1. Use of RUTS/RUSF | 1 2 3 4 5 |
|  | 1. Vitamin A supplementation for the child | 1 2 3 4 5 |
|  | 1. Use of ITNs | 1 2 3 4 5 |
|  | 1. Regular visit for Nutrition education and counselling. | 1 2 3 4 5 |
|  | 1. Overall adherence to utilization | 1. 4 or more items scoring 4-5t rating |
|  |  | 1. Less than 4 items scoring 1-3 rating |
| 4.0 | **ACCESS TO NUTRITIONAL ADVICE AND SERVICES RATING**  (Information booklet to be provided to enumerators during training)  I will read some statements here which may or may not affect you. Please give rating on your level of access to the following advice or services. | MAPPING SECTION  *Choose the most appropriate choice by circling the correct option.*  1=Strongly disagree, 2=Disagree, 3=neither disagree/agree, 4=agree, 5=Strongly agree |
| 4.1 | I have been provided very good quality services on: |  |
|  | 1. Provision and collection of IFAS | 1 2 3 4 5 |
|  | 1. Nutrition education and counseling | 1 2 3 4 5 |
|  | 1. Deworming | 1 2 3 4 5 |
|  | 1. Vitamin A supplementation for the child | 1 2 3 4 5 |
|  | 1. Sexual and reproductive health sensitive to nutrition e.g. family planning | 1 2 3 4 5 |
|  | 1. Basic environmental hygiene, and disease prevention e.g. provision of ITNs | 1 2 3 4 5 |
|  | 1. Basic personal hygiene | 1 2 3 4 5 |
|  | 1. Regular nutrition assessment both at antenatal and postnatal | 1 2 3 4 5 |
|  | 1. Child growth monitoring at postnatal care | 1 2 3 4 5 |
|  | 1. Nutrition referral for critical malnutrition episodes | 1 2 3 4 5 |
|  | 1. Nutrition support e.g. peer mother to mother support | 1 2 3 4 5 |
|  | 1. Nutrition supplements e.g. ready to use therapeutic/Supplementary foods RUTS/RUSF | 1 2 3 4 5 |
|  | 1. Regular follow-ups on utilization of services e.g. through community strategy programmes | 1 2 3 4 5 |
|  | 1. Lactation management and processes e.g. normally done using lactation charts pathways | 1 2 3 4 5 |
|  | **I have consistently received good nutrition information through/on:** |  |
|  | 1. Nutrition IEC materials e.g. brochures, leaflets etc. | 1 2 3 4 5 |
|  | 1. Nutrition information through Bulk SMS | 1 2 3 4 5 |
|  | 1. Nutrition information through Internet links referrals | 1 2 3 4 5 |
|  | 1. Nutrition information through Face to face | 1 2 3 4 5 |
|  | 1. Nutrition information through Video clips | 1 2 3 4 5 |
|  | 1. Nutrition information through Social media e.g. WhatsApp and Facebook pages | 1 2 3 4 5 |
|  | 1. Recommended food groups for expectant and lactating mother e.g. Variety depicted with at least ≤ 3 food groups (**low**), 4 and 5 food groups (**medium**), ≥ 6 food groups (**highest**) | 1 2 3 4 5 |
| 5.0 | **POWER DYNAMICS**  I will read some statements here which may or may not affect you. Please give the correct rating to reflect the true picture of your situation. | BARRIERS  *Choose the most appropriate choice by circling the correct option.*  1=Strongly disagree, 2=Disagree, 3=neither disagree/agree, 4=agree, 5=Strongly agree |
|  | **Self Esteem** |  |
|  | 1. I can resolve nutrition and dietary related problems on my own | 1 2 3 4 5 |
|  | 1. I depend on significant others to resolve nutrition and dietetics problems | 1 2 3 4 5 |
|  | 1. Whenever I want to seek nutrition services from a health facility and somebody opposes me I always push and get what I want | 1 2 3 4 5 |
|  | 1. I always get some way to deal with health and nutrition problems that confronts me. | 1 2 3 4 5 |
|  | 1. I can overcome my spouse/guardian/parents’ contrary decision to seek nutrition and health services | 1 2 3 4 5 |
|  | 1. I can take action to improve nutrition status through healthy eating during hard times | 1 2 3 4 5 |
|  | 1. My decision to seek health and nutrition services is always directed by significant others (e.g. parents, spouse, guardian, siblings) | 1 2 3 4 5 |
|  | 1. I always find it difficult to deal with health and nutrition problems that confronts me. | 1 2 3 4 5 |
|  | **Social Position** |  |
|  | 1. My spouse live with respects me and care about my nutritional health | 1 2 3 4 5 |
|  | 1. People I live with respects me and care about my nutritional health | 1 2 3 4 5 |
|  | 1. People in my community always care about my health and nutrition status | 1 2 3 4 5 |
|  | 1. People in my church always care about my health and nutrition status | 1 2 3 4 5 |
|  | 1. My father value my opinion | 1 2 3 4 5 |
|  | 1. My mother value my opinion | 1 2 3 4 5 |
|  | 1. My guardian/ value my opinion | 1 2 3 4 5 |
|  | 1. Members of extended families value my opinion | 1 2 3 4 5 |
|  | 1. My spouse/guardian/parents’ / members of extended families still show willingness to support my education despite pregnancy/lactation status | 1 2 3 4 5 |
|  | 1. My peers within the community demonstrate socio-support whenever I am in need. | 1 2 3 4 5 |
|  | 1. My guardian takes full responsibilities in educating me regardless of my status | 1 2 3 4 5 |
|  | 1. My father takes full responsibilities in educating me regardless of my status | 1 2 3 4 5 |
|  | 1. My mother takes full responsibilities in educating me regardless of my status | 1 2 3 4 5 |
|  | **Ability to decide on resource use** |  |
|  | 1. I can allocate money to seek health and nutrition services without permission from parents/spouse/guardian/siblings | 1 2 3 4 5 |
|  | 1. I am able to personally decide on which areas of my needs require resources to be allocated. | 1 2 3 4 5 |
|  | **Gender Dynamics** |  |
|  | 1. Members of this community have respect to pregnant/lactating adolescents | 1 2 3 4 5 |
|  | 1. Members of this community treat girls and boys equally | 1 2 3 4 5 |
|  | 1. Both pregnant/lactating adolescent girls and boys of same age group are given equal opportunity in youth leadership | 1 2 3 4 5 |
|  | 1. Both boys and pregnant/lactating adolescent girls of the same age group receive equal opportunity to pursue education | 1 2 3 4 5 |
|  | 1. Both boys and pregnant/lactating adolescent girls receive equal opportunity in resource allocation | 1 2 3 4 5 |
|  | 1. Cases of gender-based violence against girls who are pregnant or lactating are common in this community. | 1 2 3 4 5 |
|  | 1. Pregnant/lactating adolescent girls sometimes receive psychological, emotional and verbal abuse when they visit health facilities | 1 2 3 4 5 |

## **FGD Guide**

**Participants:**

**Community Health Workers,**

**Parents and**

**Mother-to-Mother Support Group**

**Indicators of Access to Nutritional Advice and Services**

1. Who provides nutrition advice and services for adolescent pregnant/lactating for adolescent pregnant/lactating at the health facility?
2. Mention some of the facilities nutrition pieces of advice/services provided for adolescent pregnant/lactating mothers? How accessible are these facilities?
3. How are the nutrition advise information conveyed to the adolescent pregnant/lactating whenever they visit a facility to seek services?

**Indicators of Access to Nutritional Advice and Services (Facilitator to Refer to (d) above)**

1. What is your level of satisfaction with the nutrition and health information provided to the adolescents in this community?
2. What is your level of satisfaction with the nutrition services provided to the adolescents in this community?

**Power Dynamics**

1. What are the main barriers to the access to health and nutrition services for pregnant/lactating adolescent girls?
2. Describe the extent to which the adolescent girls who are pregnant/lactating have the ability to perfume the following:
3. resolve nutrition and dietary related problems on my own
4. push and get health and nutrition needs despite opposition from significant others
5. act to improve nutrition status through healthy eating during hard times
6. What extent do members of this community accord respect to the adolescent girls who are pregnant/lactating respect they deserve on health matters? {Whichever the outcome] How does this affect adolescents’ nutrition and health status?
7. To what extent are the family members and members of this community support the adolescent girls who are pregnant/lactating g on matters of formal education?
8. What barriers are preventing adolescent girls who are pregnant/lactating to seek for services relating to their nutrition (even when they have the information)
9. What social factors prevent adolescent girls to have the right behaviors in terms of nutrition, health services and breastfeeding their babies?
10. Describe the nature of freedom for adolescent girls who are pregnant/lactating in allocating and using their resources (e.g. time, money etc.) to meet their nutrition and health needs?
11. Have you witness cases of gender-based violence among the adolescent who are pregnant and lactating? How are such cases handled?
12. What areas of inequality do things adolescent girls who are pregnant/lactating and boys of same age group treated differently?

**Recommendation**

1. What would you recommend to address the issues of nutrition and health among the adolescent pregnant/lactating?
